# The influence of weather conditions on the activity of high-arctic arthropods inferred from long-term observations

**DOI:** 10.1186/1472-6785-8-8

**Published:** 2008-05-02

**Authors:** Toke T Høye, Mads C Forchhammer

**Affiliations:** 1 Department of Arctic Environment, National Environmental Research Institute, University of Aarhus, PO Box 358 Frederiksborgvej 399, DK-4000 Roskilde, Denmark; 2 Current Address: Department of Wildlife Ecology and Biodiversity, National Environmental Research Institute, University of Aarhus, Grenåvej 14, DK-8410 Rønde, Denmark; 3 Centre for Integrated Population Ecology, www.cipe.dk, Denmark

## Abstract

**Background:**

Climate change is particularly pronounced in the High Arctic and a better understanding of the repercussions on ecological processes like herbivory, predation and pollination is needed. Arthropods play an important role in the high-arctic ecosystem and this role is determined by their density and activity. However, density and activity may be sensitive to separate components of climate. Earlier emergence due to advanced timing of snowmelt following climate change may expose adult arthropods to unchanged temperatures but higher levels of radiation. The capture rate of arthropods in passive open traps like pitfall trap integrates density and activity and, therefore, serves as a proxy of the magnitude of such arthropod-related ecological processes. We used arthropod pitfall trapping data and weather data from 10 seasons in high-arctic Greenland to identify climatic effects on the activity pattern of nine arthropod taxa.

**Results:**

We were able to statistically separate the variation in capture rates into a non-linear component of capture date (density) and a linear component of weather (activity). The non-linear proxy of density always accounted for more of the variation than the linear component of weather. After accounting for the seasonal phenological development, the most important weather variable influencing the capture rate of flying arthropods was temperature, while surface-dwelling species were principally influenced by solar radiation.

**Conclusion:**

Consistent with previous findings, air temperature best explained variation in the activity level of flying insects. An advancement of the phenology in this group due to earlier snowmelt will make individuals appear earlier in the season, but parallel temperature increases could mean that individuals are exposed to similar temperatures. Hence, the effect of climatic changes on the activity pattern in this group may be unchanged. In contrast, we found that solar radiation is a better proxy of activity levels than air temperature in surface-dwelling arthropods. An advancement of the phenology may expose surface-dwelling arthropods to higher levels of solar radiation, which suggest that their locomotory performance is enhanced and their contribution to ecological processes is increased.

## Background

The earth is undergoing climatic changes and the model predictions for the high-arctic climate are particularly dramatic [1]. This underscores the urgency for achieving a better understanding of how climate affects ecological processes like herbivory, predation and pollination [2]. The major controls of such processes are the population size of the functional group (e.g. herbivores, predators or pollinators) and the activity level of the individuals. A particularly strong response to climatic conditions can be expected in high-arctic arthropods since they are poikilothermic and because the abiotic conditions of the High Arctic are generally close to the minimum requirements for locomotion [3]. The seasonal emergence of adult arctic arthropods (i.e. their phenological development) is determined mainly by the timing of snowmelt [4-7], whereas their activity pattern is a function of body temperature. The microclimatic conditions that determine body temperature in arthropods is an integration of a suite of weather variables (e.g. radiation, precipitation and wind speed) in addition to temperature [8-11]. Hence, the magnitude of arthropod-related ecological processes in the High Arctic depends on several potentially uncorrelated components of climate. We have previously shown that high-arctic arthropods have advanced their emergence phenology in recent years in response to earlier timing of snowmelt [12]. This shift in phenology may expose arthropods to a time of the season with different weather conditions and may therefore affect their level of activity.

Capture rates from passive traps like pitfall traps integrate arthropod density and activity levels [13] and therefore provides a proxy for the magnitude of the ecological processes in which the organisms are involved. For instance, the capture rate of wolf spiders is related to their level of activity as well as their density. As such it is a correlate of the predation pressure by wolf spiders. Likewise, the capture rate of pollinators provides a proxy for the magnitude of pollination. The effect of weather on activity patterns is immediate whereas the physiological processes controlling density involves emergence and mortality. Hence, the time scale of fluctuations in activity is likely to be shorter than the time scale of fluctuations in density. Previous work from seasonal environments has shown that these two factors can be separated statistically [14]. Here, we extend this technique by applying generalized additive models [15]. Using this modelling approach, it is possible to separate the variation in capture rate due to a component which varies over a short time scale (activity) from variation due to a component which varies over a longer time scale (density) without *a priori *assumptions about its exact shape. We aim at elucidating broader patterns in the relation between climate and arthropod ecology, and therefore we take a higher taxon approach. We addressed the following questions: Which meteorological variable most strongly influences the locomotory performance of different taxa of flying and surface-dwelling arthropods in a high-arctic location? What is the relative influence of weather parameters and capture date on capture numbers of these taxa?

## Methods

### Study area and data

**Figure 1:**
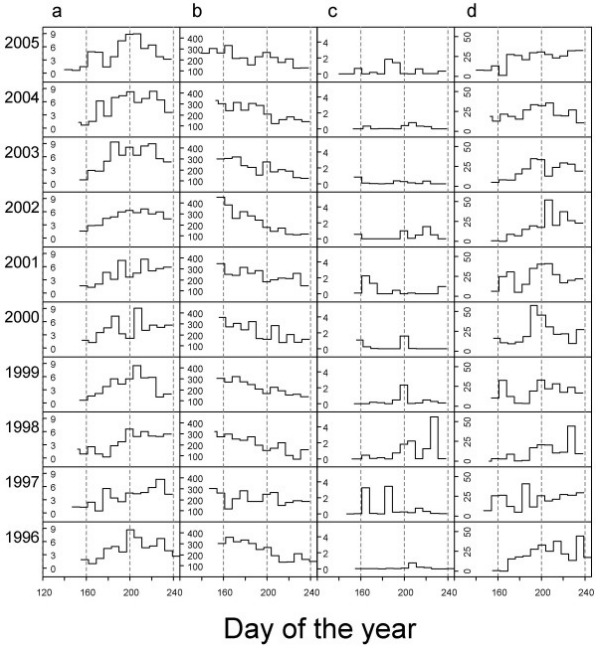
**Inter-annual dynamics of weather variables**. Variation in weather variables calculated for each of the trapping periods in each year during 1996–2005. a) Thawing day-degrees (°C/day), b) Incoming short-wave radiation (W/m^2^), c) Average daily precipitation (mm/day) and d) Frequency of high wind speeds indexed by the number of 10 min intervals with wind speed above 3 m/s per day.

The data for this study was collected as part of the Zackenberg Basic monitoring programme. The sampling was carried out at Zackenberg, North-east Greenland (74°28'N; 20°34'W) which is in the high-arctic climatic zone. The mean summer (June through August) temperature during the years 1996–2005 was 4.3°C. At Zackenberg, June, July and August include on average 85% (range: 73%–93%) of the annual number of days with positive average temperatures. A climate station within 600 metres from all sampling plots provided data on ambient air temperature two metres above the ground, solar radiation (W/m^2^, SR), precipitation (mm, PREC) and wind speed (m/s) [16]. Wind speed was recorded every ten minutes and other weather variables were recorded hourly throughout the study period [17]. We calculated thawing degree days (TDD) by letting the daily contribution to TDD equal the mean of all hourly air temperature measurements where recordings of subzero temperatures were set to zero. To accommodate for the anticipated non-linear response of arthropods to wind speed, wind data were converted to the number of ten minute intervals per day with wind speed higher than 3 m/s (WIND, Fig. 1).

Arthropods were monitored during 10 consecutive years (1996–2005) with samples from one window trap plot (plot 1) and six pitfall trap plots (plot 2–7) collected weekly during June, July and August. All plots were operated during the period 1996–2005 except for plots 6 and 7 which were operated during the periods 1996–1998 and 1999–2005, respectively. Each pitfall trap plot (10 × 20 m^2^) consisted of eight pitfall traps and each window trap plot consisted of two window traps [18]. The window trap plot was located next to a pond. The pitfall traps were yellow plastic cups 10 cm in diameter and each window trap consisted of two chambers and the two traps were placed perpendicular to each other [see [19] for details]. Trapping started in June once the snow at each trap had melted. All specimens caught in all years were sorted to the family level except for collembolans and mites who were only counted. Here, we focus on: Chironomidae (Diptera), Muscidae (Diptera), Sciaridae (Diptera), Ichneumonidae (Hymenoptera), Nymphalidae (Lepidoptera), Lycosidae (Aranea), Linyphiidae (Aranea), collembolans and mites. The family Nymphalidae is represented by two species (*Boloria chariclea*, (Schneider) and *B*. *polaris*, (Boisduval)), the Lycosidae by one species (*Pardosa glacialis*, (Thorell)) and the Linyphiidae by five species (*Collinsia thulensis*, (Jackson), *Hilaira vexatrix*, (O. P.-Cambridge), *Erigone arctica *(White), *Erigone psychrophila*, Thorell and *Mecynargus borealis*, (Jackson)) [20]. The data set included a total of 531,036 individuals, each belonging to one of the nine taxa. This corresponds to 93.6% of all arthropod specimen caught during the study period. Traps were occasionally flooded, emptied by arctic foxes (*Alopex lagopus*, (Linneaus)), or trampled by muskoxen (*Ovibos moschatus*, (Zimmermann)), so the capture numbers in each plot were transformed to individuals caught per trap per day for each trapping period [7].

### Statistical analyses

Our aim was to model the concurrent influences of ambient weather and arthropod phenology on capture numbers. Since we anticipated a non-linear phenological development through each season but had no *a priori *assumptions about its exact shape, we used partial smoothing splines in generalized additive models (GAM) [21] to model the seasonal development in capture rates. This family of models identifies the most likely relationship between parameters based on a non-parametric back-fitting algorithm [15] and so is particularly useful in situations where a non-linear relationship is anticipated but its form is unknown. We developed full models of the following general structure:

Y_*i *_= β_*0 *_+ s(DAY) + β_*1 *_× WEATHER + ε_*i*_

Where Y_*i *_are the log_10_-transformed number of individuals per trap per day, β_0 _is the intercept and trapping date (DAY) is modelled by a spline function s(·). The term WEATHER refers to one of the four different weather variables (TDD, SR, PREC and WIND) and was modelled as a linear continuous predictor and ε_*i *_is the error term. The weather variables were calculated as daily averages for each trapping period. We repeated the model estimation for each species group (n = 9) in each plot (n = 7 for flying arthropods, n = 6 for surface-dwelling arthropods, because this group was excluded from the window trap plot) in each year (n = 10) with each of the weather parameters (n = 4) as linear predictors and date as a spline function (with df = 4). This resulted in a total of 500 sets of four competing models. Some taxa were only caught in small numbers in some plots in some years, and we therefore restricted our analyses to years and plots where at least 100 individuals were caught except for the Nymphalidae and the Ichneumonidae where the limit was set to 50 due to the larger size of individuals in these families. This reduced the number of sets of models to 385. We had an average of 11.5 trapping periods within each season. Although it is likely that the capture numbers were affected by several weather variables, we included only one in each model to reduce the risk of over-parameterization. Since all models with the same response variable had the same number of degrees of freedom and the same null deviance, the lowest residual deviance indicated the best fit to data. In this way, we identified for each species group for each plot for each year the weather variable that was best able to explain variation in capture numbers. A previous study [14] separated seasonal patterns of abundance from activity by first fitting a Gaussian curve to the data and using weather variables to explain variation in the residuals. In addition to fitting GAM models we adopted this approach. Hence, we fitted Gaussian curves to the entire set of 500 time series and identified the weather variable that best explained variation in the residuals from linear regression models.

## Results

The GAM models identified the weather variable that resulted in the lowest residual deviance of models for each species group in each year in each plot (Table 1). Ambient air temperature was best at explaining variation in capture numbers followed by solar radiation which was particularly important in models of surface-dwelling taxa. The weather parameter that explained most of the variation was mostly the same in both data sets and across plots, but in a few cases there were differences among trap types. However, in all cases the final reduced models included the smoothing spline of capture date. Thus, there was a non-linear effect of capture day in all species for both trap types (Table 1). WIND and TDD were significantly correlated reflecting parallel seasonal development of wind and temperature regimes. In eight out of twelve cases (taxa and trap types combined) the GAM modelling and the use of Gaussian curves identified the same weather variable as the most important.

**Table 1:** Statistical analysis of capture data in relation to weather patterns

**Taxon**	**Weather variable in best model**				**Number of models**	**Average explained deviance in %**		
			
	**SR**	**TDD**	**WIND**	**PREC**		**s(·)**	**Weather**	**Final model**
**Window traps**								
Chironomidae*	20.00	27.00	**30.00**	23.00	10	79.67	7.52	85.81
Muscidae*	27.00	**30.00**	19.00	24.00	10	85.74	23.47	89.68
Sciaridae*	16.67	**33.33**	26.67	23.33	3	58.91	18.26	80.69
**Pitfall traps**								
Chironomidae	24.67	**28.89**	25.11	21.33	45	82.64	12.91	88.56
Muscidae	**28.80**	26.80	21.80	22.60	50	84.38	15.58	90.87
Sciaridae*	25.14	**31.14**	23.43	20.29	35	78.15	16.10	85.82
Nymphalidae*	26.11	**31.67**	26.11	16.11	18	76.26	29.22	90.28
Ichneumonidae	**26.19**	25.71	**26.19**	21.90	42	81.66	19.45	86.65
Linyphiidae*	**35.00**	31.67	-	33.33	30	75.98	19.21	83.06
Lycosidae*	**42.20**	30.85	-	26.95	47	70.70	24.51	85.48
Acari	34.07	**35.56**	-	30.37	45	78.53	23.22	89.28
Collembola*	33.00	32.67	-	**34.33**	50	71.88	13.82	81.09

Summary results of generalized additive models of the ten years of data for the different arthropod taxa aggregated across years and plots. Models with each of four weather variables: solar radiation in W/m^2 ^(SR), thawing day-degrees in °C (TDD), proportion of capture period with wind speeds above 3 m/s (WIND) or precipitation in mm (PREC) were ranked from one to four, with four assigned to the model with the best fit. The table gives the summed rank relative to the possible maximum (in %) for each of the four weather variables. The weather variable that was ranked highest is given in bold for each taxon in each of the two trap types. In addition, the number of sets of models is given as well as the average percentage of null deviance explained by the generalized additive models of a spline of capture date alone s(·), of the weather variable alone and finally of the combined model of both the spline of capture date as well as the linear weather variable. Asterisks indicate that the use of Gaussian curves (parametric models) instead of GAM's identified the same weather variables as the most important.

## Discussion

Our analyses demonstrated that the temporal variation in capture rates of high-arctic arthropods from pitfall and window traps consisted of two components indicating the effects of density and activity. By using generalized additive modelling to separate variation in capture rates, we found one component that was unimodally related to capture date and one that was a function of short-term weather fluctuations through the season. Air temperature was the most important weather variable followed by solar radiation. The influence of temperature was most pronounced on the taxa of flying arthropods like Lepidoptera and Diptera, which is consistent with previous findings [14]. The surface-dwelling taxa like the spiders (Linyphiidae and Lycosidae) responded most strongly to variation in solar radiation. Solar radiation is probably a better predictor of near-surface temperatures than ambient air temperature two metres above the ground. Although, capture rates may be influenced by factors other than density and activity, such as seasonally-determined changes in behaviour related specifically to reproduction, our flexible non-linear functions would have included such effects in the function of capture date. When we reran the entire analysis using Gaussian curves to model the seasonal development the same weather variables were identified as the most important in eight of twelve cases. The deviation between the model approaches could arise if the seasonal development does not follow a Gaussian curve. Alternatively, the spline function of the GAM could have captured some of the variation due to variation in activity levels, but with the degrees of freedom in the spline (i.e. the flexibility of the curve) set at a constant and low level this is rather unlikely. The good correspondence between the two methods lends further support to the conclusion that the identified weather variables are indeed affecting the activity level of the individual species groups.

Species-specific responses may be hidden by pooling multiple species in larger taxonomic units. However, we found no bimodality in the seasonal capture of the taxa included in this study which could suggest that individual species within a taxon occurred during different parts of the season. Also, since flying and surface-dwelling arthropods responded to different components of climate, general differences in the environmental conditions experienced by these two groups may be more important than behavioural differences between species within taxa. Clearly, this higher taxon approach precludes a detailed understanding of the species-environment interactions. However, since our aim here is to elucidate broader patterns in the relation between climate and arthropod ecology, we refer to these interactions simply as arthropod-related ecological processes. It is possible that sampling with shorter time intervals would have identified a stronger influence of weather variables on activity of the arthropods. However, without increasing the number of traps this would also have reduced the sample size from each sampling and thereby reduced the statistical power of the analyses. Indeed we are confident that by using data from ten seasons we identified the most important weather variable for the activity level of each taxon.

Our non-linear statistical modelling approach also made it possible to quantify the relative importance of density and activity. It has long been acknowledged that passive traps like pitfall traps provide biased estimates of density, because differences in activity levels between trapping periods are difficult to control for [22]. It has stimulated the development of alternative sampling techniques to provide unbiased estimates of population density [23,24]. However, in some cases no alternative sampling technique is feasible. Indeed, some organisms are simply too small and fragile for applying mark recapture techniques or too mobile, cryptic or non-randomly distributed to allow for counting using a transect approach. In these cases, parallel data on dominating meteorological variables make it possible to control for the effect of differences in locomotory performance among trapping periods. Also, capture rates in passive traps may actually provide a proxy for the rate of ecological processes involving arthropods.

**Figure 2:**
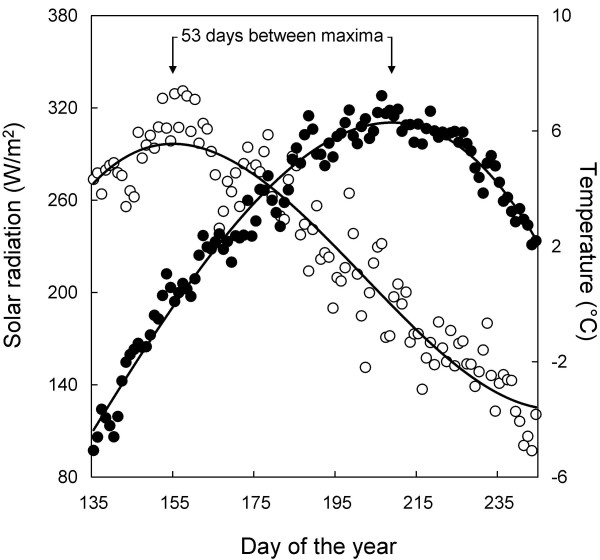
**Seasonal dynamics of solar radiation and temperature**. The level of average daily incoming solar radiation (open dots) and temperature (filled dots) in relation to day of the year averaged for the years 1996–2005. A third-order polynomial fit to the temperature and solar radiation data is indicated by a line and the distance between maxima of the two models is given in days.

We found that the non-linear component of the general additive models representing density explained more of the variation than did the weather variables representing locomotory performance. This indicates that the magnitude of arthropod-related ecological processes is currently more influenced by phenological changes than by short-term fluctuations in weather. However, the dynamics of the two most important weather variables (temperature and solar radiation) differed markedly across the season and therefore a change in the timing of the phenology of the species groups could also increase the importance of weather on locomotory performance. The summer peak of incoming solar radiation occurred almost two months earlier than the summer peak in average air temperature (Fig. 2). Since taxa of flying arthropods are mostly sensitive to variation in air temperature and taxa of surface-dwelling arthropods are mostly sensitive to variation in solar radiation, changes in phenological timing may have opposite effects on these two groups of organisms. We have previously shown that arthropod phenology at Zackenberg is more closely linked to timing of snowmelt than to temperature for the taxa included in this study [7]. Consequently, in species where the activity level is most sensitive to temperature, an advancement of the timing of snowmelt would result in an unchanged or decreased contribution to ecological processes, depending on the magnitude of the parallel temperature increase. In contrast, species where the activity level is mostly influences by solar radiation would probably contribute more to ecological processes by an advancement of their emergence, because they would be present during a period with higher levels of solar radiation.

## Conclusion

We were able to statistically separate the variation in capture rates of high-arctic arthropods into a non-linear component of capture date (density) and a linear component of weather (activity). Consistent with previous findings, air temperature best explained variation in the activity level of flying insects. An advancement of the phenology in this group due to earlier snowmelt will make individuals appear earlier in the season, but parallel temperature increases could mean that individuals are exposed to similar temperatures. Hence, the effect of climatic changes on the activity pattern in this group may be unchanged. In contrast, we found that solar radiation is a better proxy of activity levels than air temperature in surface-dwelling arthropods. An advancement of the phenology may expose surface-dwelling arthropods to higher levels of solar radiation, which suggest that their locomotory performance is enhanced and their contribution to ecological processes is increased. Therefore, the integrated effect of climatic changes on ecological processes depends on the change in both temperature and timing of snowmelt and may vary between processes involving surface-dwelling and flying arthropods.

## Authors' contributions

TTH and MCF conceived the idea TTH carried out the analyses and drafted the manuscript. Both authors have read and approved the final manuscript.

## References

[B1] Kattsov VM, Källén E, Cattle H, Christensen J, Drange H, Hanssen-Bauer I, Jóhannesen T, Karol I, Räisänen J, Svensson G, Vavulin S (2005). Future climate change: modeling and scenarios for the Arctic. Arctic Climate Impact Assessment.

[B2] Walther GR, Post E, Convey P, Menzel A, Parmesan C, Beebee TJC, Fromentin JM, Hoegh-Guldberg O, Bairlein F (2002). Ecological responses to recent climate change. Nature.

[B3] Strathdee AT, Bale JS (1998). Life on the edge: insect ecology in arctic environments. Annu Rev Entomol.

[B4] Danks HV (1978). Some effects of photoperiod, temperature, and food on emergence in three species of Chironomidae (Diptera). Can Entomol.

[B5] MacLean SFJr, Pitelka FA (1971). Seasonal patterns of abundance of tundra arthropods near Barrow. Arctic.

[B6] Corbet PS, Danks HV (1973). Seasonal emergence and activity of mosquitoes (Diptera: Culicidae) in a High-Arctic locality. Can Entomol.

[B7] Høye TT, Forchhammer MC (2008). Phenology of high-arctic arthropods: effects of climate on spatial, seasonal and inter-annual variation. Adv Ecol Res.

[B8] Peng RK, Fletcher CR, Sutton SL (1992). The effect of microclimate on flying dipterans. Int J Biometeorol.

[B9] Edde PA, Phillips TW, Nansen C, Payton ME (2006). Flight activity of the lesser grain borer, *Rhyzopertha dominica *F. (Coleoptera: Bostrichidae), in relation to weather. Environ Entomol.

[B10] Waringer JA (1991). Phenology and the influence of meteorological parameters on the catching success of light-trapping for Trichoptera. Freshwater Biol.

[B11] Service MW (1980). Effects of wind on the behaviour and distribution of mosquitoes and blackflies. Int J Biometeorol.

[B12] Høye TT, Post E, Meltofte H, Schmidt NM, Forchhammer MC (2007). Rapid advancement of spring in the High Arctic. Curr Biol.

[B13] Southwood TRE, Henderson PA (2000). Ecological methods.

[B14] Briers RA, Cariss HM, Gee JHR (2003). Flight activity of adult stoneflies in relation to weather. Ecol Entomol.

[B15] Hastie TJ, Tibshirani RJ (1990). Generalized additive models.

[B16] Meltofte H, Thing H (1996). Zackenberg Ecological Research Operations 1st Annual Report 1995.

[B17] Rasch M, Caning K (2005). Zackenberg Ecological Research Operations, 10th Annual Report, 2004.

[B18] Jónsson E, Gardarsson A, Gíslason G (1986). A new window trap used in the assessment of the flight periods of Chironomidae and Simuliidae (Diptera). Freshwater Biol.

[B19] Meltofte H, Berg TB (2006). BioBasis – Conceptual design and sampling procedures of the biological programme of Zackenberg Basic.

[B20] Larsen S, Caning K, Rasch M (2001). Diversity and composition of the spider fauna in different vegetation types at Zackenberg. Zackenberg Ecological Research Operations, 6th Annual Report, 2000.

[B21] Venables WN, Ripley BD (2002). Modern applied statistics with S.

[B22] Topping CJ, Sunderland KD (1992). Limitations to the use of pitfall traps in ecological studies exemplified by a study of spiders in a field of winter wheat. J Appl Ecol.

[B23] Perner J, Schueler S (2004). Estimating the density of ground-dwelling arthropods with pitfall traps using a nested-cross array. J Anim Ecol.

[B24] Thomas CFG, Parkinson L, Marshall EJP (1998). Isolating the components of activity-density for the carabid beetle *Pterostichus melanarius *in farmland. Oecologia.

